# Risk of congenital birth defects during COVID-19 pandemic: Draw attention to the physicians and policymakers

**DOI:** 10.7189/jogh.10.020378

**Published:** 2020-12

**Authors:** Md Sakirul Islam Khan, Hiroaki Nabeka, Sheikh Mohammad Fazle Akbar, Mamun Al Mahtab, Tetsuya Shimokawa, Farzana Islam, Seiji Matsuda

**Affiliations:** 1 Department of Anatomy and Embryology, Ehime University Graduate School of Medicine, Toon, Ehime, Japan; 2 Department of Gastroenterology and Metabology, Ehime University Graduate School of Medicine, Toon, Ehime, Japan; 3 Department of Hepatology, Bangabandhu Sheikh Mujib Medical University, Dhaka, Bangladesh

**Figure:**
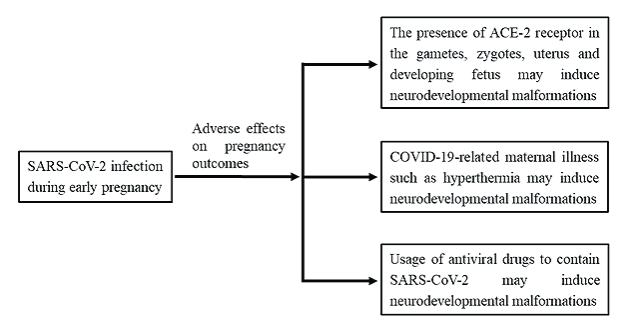
Photo: COVID-19 in pregnant women and possible risk to develop congenital birth defects.

The coronavirus disease 2019 (COVID-19), caused by the severe acute respiratory syndrome coronavirus 2 (SARS-CoV-2), represents a global public health emergency with considerable morbidity and mortality. Since its first description in late 2019, SARS-CoV-2 has already spread worldwide. As of mid-May 2020, over 4.5 million people have already been infected globally by SARS-CoV-2 with more than 300 000 deaths [1]. It is almost clear that SARS-CoV-2 has infected both male and female in almost same proportions. Although every human is susceptible to the infection, data show that relatively more female has been infected at their active reproductive age (between 20 years to 49 years) [2]. Therefore, it is likely that numerous females during their pregnancy have already been infected with SARS-CoV-2. As the world is in the middle of a pandemic, the majority efforts have been attributed to innovation of anti-COVID therapy for management of patients and development of prophylactic vaccines for SARS-CoV-2 so that transmission cycle of the virus can be contained. However, little has been explored about SARS-CoV-2 infection and the implications of anti-COVID-19 drugs on pregnancy.

Members of the coronavirus family are known to be responsible for severe complications during pregnancy, such as miscarriage, fetal growth restriction and congenital anomalies [3]. Only few studies to date have reported, relatively higher rates of adverse birth outcomes in women affected by SARS-CoV-2 at late pregnancy [3]. Considering the possible adverse effects of SARS-CoV-2 infection in early pregnancy, the American Society of Reproductive Medicine as well as other similar professional organizations recommend individuals with confirmed or presumed COVID-19 patients should avoid pregnancy or not to have fertility treatments during outbreak of COVID-19. However, this seems to be unrealistic as most of the scientific information will not reach the huge population of developing and resource-constrained countries. On the other hand, there is potential chance of increased conception due to stay home and other approaches such as lockdown situation in many countries as people have been confined to their houses. These realities indicate that the medical community should have a clear conception about the implications of SARS-CoV-2, COVID-19 and the anti-COVID drugs on early pregnancy when some intractable neurological complications develop. In this perspective, we would intend to provide an outline of the role of SARS-CoV-2, COVID-19 and the role of antiviral drugs (using for containment of COVID-19) on neural tube defects (NTDs), one of the severe congenital malformations develop in early pregnancy, to draw attention of physicians and policymakers to this formidable challenge.

Published literatures have indicated that viral illness during early pregnancy and several antiviral drugs are associated with an increased risk for neurodevelopmental congenital anomalies of newborn [4]. These include NTDs, the most common and severe malformations of spinal cord (spina bifida) or brain (anencephaly, encephalocele, hydrocephalus), which develop within 6 weeks of pregnancy with an incidence of one in 1000 neonates worldwide and cause lifelong neurological complications [5]. NTDs are leading causes of pediatric hospitalization, medical expenditure, and infant mortality. In fact, it is estimated that NTDs resulting approximately 88 000 neonatal deaths (29% in low-income countries) and 8.6 million disability adjusted life years [6]. In addition to enormous suffering of the patient, the public health and social impact of these diseases are also extremely notable as an estimated lifetime medical costs exceeds US$81.00 million per year for children born with spina bifida [5]. Since viral illness and its therapeutic approaches are associated with NTDs in infants [4], there may be some long-lasting health burden related to SARS-CoV-2 infection during pregnancy, and these have remained unnoticed till now.

Scientific evidences indicate that the causative agent of COVID-19, SARS-CoV-2 seem to cross both placental barrier (viral IgM detected in infants hours after birth) [7] and blood brain barrier (virus detected in cerebrospinal fluid) [8]. As the virus can enter placenta and nervous system, the virus itself may have some adverse effects on the pathogenesis of NTDs, if pregnant mothers suffer from COVID-19. Also, it appears that coronavirus, SARS-CoV-2, may be transmitted to fetus from mother as the virus use entry receptor, angiotensin-converting enzyme 2 (ACE2) and S protein proteases expressed in developing human embryo. Notably, ACE2 and S protein proteases are expressed in early gametes, zygotes, and 4-cell embryos [9]. Thus, direct transmission of infection of blast cells by SARS-CoV-2 may be possible, but remains to be confirmed. In developing embryos, the health of these cells of the epiblast is crucial as these cells undergo organogenesis. Any functionally alterations in early embryonic cells by the viral infection may lead to adverse birth defects. With much still unknown about COVID-19 and neurodevelopmental complications, there is an increased risk to develop congenital birth defects, if SARS-CoV-2 infection occurs during early pregnancy. However, the severity of COVID-19 pandemic has disrupted the normal process of patient counseling, case compilation and data processing at present. There is, therefore, an urgent need to continue collecting data on clinical cases of COVID-19 infection in pregnancy particularly during first or early second trimester, and to improve our understanding regarding the role of COVID-19 on NTDs.

Next, there are several concerns about the usage of antiviral drugs to contain SARS-CoV-2, and control virus-related complications, such as pneumonia. Although no effective drug has been developed to contain SARS-CoV-2, some antiviral drugs as well as anti-inflammatory agents developed for other viral infections and pathologies are widely used in COVID-19 patients around the world. Many of these drugs have been used for COVID-19 without undergoing proper safety and efficacy tests as we are now facing a serious pandemic. The usage of hydroxychloroquine is a controversial issue at present as this drug has been reported to cause many complications including death. Although chloroquine is classified as class C in the US Food and Drug Administration (FDA) for pregnancy, this drug is now widely used for COVID-19 treatment around the world.

Similarly, drugs like favipiravir, a drug developed to treat influenza virus disease; remdesivir to treat Ebola virus disease; and dolutegravir/lamivudine/tenofovir to treat human immunodeficiency viruses (HIV) have been used for COVID-19 treatment around the world on the basis of their availability. In fact, some of these drugs have been given emergency licensing and approval by US FDA. As the effects of these drugs have not been checked in pregnancy particularly in early trimester, there is growing fear suggesting that antiviral drugs may cause adverse birth outcomes. Favipiravir is contraindicated in women who might be or are pregnant because of its association with birth defects [10], however, this drug is now widely being used to treat COVID-19 in about 40 countries with the assistance of Japanese government and also in several developing countries including Bangladesh as a trial drug received from their own source. The drug has not been recommended by US FDA or Japanese Pharmaceuticals and Medical Devices Agency (PMDA), however, the availability of the drug and possible effects in COVID-19 has made a drug of choice or a golden bullet. Dolutegravir, an effective antiretroviral therapy for HIV treatment, is promising choice in low-income and middle-income countries, is also used for COVID-19 treatment. However, recent findings reveal that dolutegravir increases number of NTDs; the prevalence of NTDs is 3 times higher with dolutegravir [11]. Also, the effect of dolutegravir on external structural abnormalities of infants has been documented at high percentage (9 per 1000 births) if this drug is used during conception [11].

Taken together, as of today, the speed of designing and initiating trials to evaluate potential COVID-19 therapeutics is really impressing, as expected. However, less impressive is the fact that due importance and consideration has not been given about one of the most important fact; the effects of the virus and management strategy on pregnant women particularly those are in early pregnancy. Now, world is in middle of pandemic and COVID-19 would prevail for at least one or more years. Thus, abstinence from being pregnant is not a practical option. Rather, it is needed to check the pregnancy state of COVID-19 patients with an eye regarding adverse pregnancy outcomes including NTDs. Also, use of antiviral drugs should be regulated in COVID-19 pregnant patients, particularly those are in early pregnancy, until its safety and potential efficacy is not ascertained for neonates by randomized clinical trial.

In conclusion, it seems that COVID-19 may results in long-lasting congenital anomalies of infants either by infection or by therapeutic maneuver. These realities become more relevant in developing and resource-constrained countries where the screening before birth particularly at early pregnancy is almost non-existing. However, the incidence and prevalence of COVID-19 is not low in these countries. Also, another spectrum should be considered at this time. Pregnancy is monitored by obstetrician and gynecologists, whereas COVID-19 is managed by virologists and infectious disease specialists. Therefore, intradepartmental collaboration and exchange of information about management and treatment of COVID-19 and NTDs are required for avoiding disastrous complications in neonates.
